# Hydrogel-Based Finger Foods: Enhancing Nutritional Intake and Swallowing Safety in Older Persons with Dysphagia

**DOI:** 10.3390/nu17203289

**Published:** 2025-10-20

**Authors:** Enrika Lazickaitė, Milda Keršienė, Viktorija Eisinaitė, Ina Jasutienė, Gytė Damulevičienė, Daiva Leskauskaitė

**Affiliations:** 1 Department of Food Science and Technology, Kaunas University of Technology, LT-50254 Kaunas, Lithuania; enrika.lazickaite@ktu.lt (E.L.); milda.kersiene@ktu.lt (M.K.); viktorija.eisinaite@ktu.lt (V.E.); ina.jasutiene@ktu.lt (I.J.); daiva.leskauskaite@ktu.lt (D.L.); 2 Department of Geriatrics, Lithuanian University of Health Sciences, LT-44307 Kaunas, Lithuania; 3 Geriatrics Centre, Lithuanian University of Health Sciences Kaunas Hospital, LT-47144 Kaunas, Lithuania

**Keywords:** older adults, nutrition, dysphagia, agar–carboxymethylcellulose composite hydrogels, water delivery, vitamin release, finger foods

## Abstract

Background: Dysphagia is a common problem in older adults, characterized as a swallowing disorder that prevents food from passing from the mouth to the esophagus. Besides impairing dietary intake and leading to malnutrition, dysphagia also severely restricts water intake. Purpose: This study aimed to develop polysaccharide-based hydrogels as dysphagia-friendly finger foods designed to provide high water content and enable controlled vitamin delivery to older persons with dysphagia. Procedures: Agar–carboxymethylcellulose (Agar-CMC) composite hydrogels with incorporated vitamins C, B_9_, B, and D_3_ were developed and tested for their textural and rheological properties, vitamin stability during storage, and vitamin release under simulated gastrointestinal conditions. Finally, a fiberoptic endoscopic swallowing assessment and sensory evaluation were conducted. Main Findings: Increasing the agar concentration in Agar-CMC hydrogels improved their internal structure and handling properties as finger foods, while still being easily breakable during swallowing. Agar-CMC hydrogels’ structure protected vitamins during processing and six weeks of storage. Vitamin release started immediately and remained steady in the gastric phase, with a noticeable increase at the beginning of the intestinal phase, resulting in 70–100% vitamin release by the end of this phase. The Fiberoptic Endoscopic Swallowing Evaluation confirmed their suitability for individuals with mild to moderate oropharyngeal dysphagia, with a low risk of aspiration (1 point on the Penetration-Aspiration Scale out of 8). Principal Conclusions: The developed Agar-CMC hydrogels present a promising dysphagia-friendly finger food alternative with high water content. They effectively deliver essential vitamins throughout the gastrointestinal tract, and notably demonstrate a low aspiration risk, making them suitable for individuals with mild to moderate oropharyngeal dysphagia.

## 1. Introduction

Dysphagia is a condition that affects the safety of swallowing and feeding for those who have it [1]. The main complication of dysphagia is aspiration pneumonia, which is an inflammation of the lungs that happens when solid or liquid food enters the respiratory tract. Besides impairing dietary intake, which can lead to malnutrition, dysphagia also significantly restricts water intake [2]. Using thickened liquids is an effective treatment to lower the risk of respiratory tract invasion in patients with dysphagia. Scientific research also supports the beneficial impact of thickened liquids on the hydration status of patients with dysphagia [3]. However, some studies suggest that only consuming thickened liquids does not substantially increase the body water content of these patients [4]. Older adults in nursing homes who require a modified-texture diet tend to drink much less fluid compared to those who consume regular fluids [5].

Psychological issues such as depression, isolation, cognitive impairment, dementia, and the inability to eat and drink independently are also common among the older population. Studies show that Alzheimer’s patients prefer foods they can eat with their fingers instead of using cutlery when it becomes too difficult to manage [6]. “Finger foods” refers to small, easily manageable food items that can be eaten without utensils and picked up with one hand [7]. Nutrition and geriatrics experts indicate that food specially prepared to be eaten with the fingers, easily grasped and moved from the plate to the mouth, can benefit older individuals with cognitive disorders. Studies are being published that demonstrate the positive effect of finger food on food consumption, particularly among individuals with dementia [8].

Another aspect of the diet among older adults is a reduced intake of fruits and vegetables [9], which are essential sources of micronutrients. A systematic review of 37 studies involving over 28,000 older individuals revealed that many older men and women had inadequate intakes of various micronutrients, particularly thiamine, riboflavin, vitamin D, calcium, magnesium, and selenium, compared to the recommended daily levels [10]. Deficiencies in micronutrients like iron (Fe), calcium (Ca), selenium (Se), vitamins C and D, vitamins B_6_ and B_12_, folic acid, and the trace element zinc (Zn) are closely associated with impaired immune function [11]. Research has also found a link between dementia (including Alzheimer’s disease and other types) and low levels of folate, vitamin B_12_, vitamin E, vitamin C, vitamin D, selenium, omega-3 fatty acids, and choline [12].

In our study, we aimed to demonstrate that polysaccharide hydrogels can be effectively utilized in the nutrition of older individuals with dysphagia to enhance both nutritional and water intake while maintaining swallowing safety. Polysaccharides are biopolymers composed of many repeating monomer units linked together [13]. Hydrogels derived from polysaccharides are three-dimensional polymer networks connected by covalent cross-links or weaker forces such as hydrogen or ionic bonds, which give gels their characteristic elasticity. These hydrophilic polymer materials can absorb and hold up to 99% of their weight in water within their gel structure [14]. Polysaccharide hydrogels, whether used alone or in combination with other biomacromolecules, can serve as delivery systems for micronutrients [15] and vitamins [16,17], providing controlled release and enhanced bioavailability. When exposed to specific environmental conditions (e.g., pH, ionic strength, temperature, enzymes), hydrogels can break down, which is useful for the targeted delivery of bioactives [18].

Addressing the risk of dehydration in patients with dysphagia, this study aimed to develop polysaccharide-based hydrogels as dysphagia-friendly finger foods with high water content and controlled vitamin delivery. A rheologically responsive approach was used to characterize the behavior of agar–carboxymethylcellulose composite hydrogels during swallowing. The suitability of the formulated hydrogels for vitamin delivery and their controlled release were evaluated throughout the gastrointestinal tract. Finally, the sensory acceptability and swallowing safety of the agar–carboxymethylcellulose composite hydrogels were assessed.

## 2. Materials and Methods

### 2.1. Preparation of Agar-CMC Hydrogels

Dry raw materials were weighed and thoroughly mixed in the following proportions: agar (0.8%, 1.0%, or 1.5% by mass), sodium carboxymethylcellulose (0.5% by mass), sodium benzoate (0.05% by mass), and potassium sorbate (0.1% by mass); chemicals were from EUROCHEMICALS, Vilnius, Lithuania;. The concentrations of agar and sodium carboxymethylcellulose in this experiment were selected based on the results of previous studies, selecting composite hydrogels that retained their shape and did not break into small pieces when dissolved in the mouth. Hydrogels were prepared by mixing and homogenizing the dry mixture with water (71–78% by mass) and fruit juice concentrate (18–27% by mass). The pH of the resulting solution was measured with a pH meter and adjusted to 3.1–3.2 using citric acid. The solution was heat-treated at 98–100 °C for up to 5 min to dissolve the components. It was then cooled to 54–56 °C. Vitamins C (0.15% by mass) obtained from the Myprotein Co. (The Hut Group, Cheshire, UK), B_9_ (0.0006% by mass), B_12_ (0.004% by mass), and D_3_ (0.016% by mass) obtained from the DSM Nutritional Products Ltd. (Basel, Switzerland) were weighed and thoroughly homogenized into the solution, which was then poured into molds and stored at 2–6 °C until gelation. After 1 h, the solidified hydrogels were demolded, placed into plastic boxes, stored at +4 °C, and prepared for further analysis. For the vitamin stability during storage, hydrogels were stored at 4 °C for 6, 16, and 24 weeks.

### 2.2. Texture Profile Analysis (TPA)

Texture profile analysis (TPA) was conducted using a texture analyzer (TA.XT Plus, Stable Micro Systems Ltd., Godalming, UK). The analysis used the P/20 (20 mm aluminum cylinder) measurement system. Tests were performed at a speed of 1 mm s^−1^, compressing the sample to 50% of its height, with a 5 s delay between two compressions. Force–time curves were automatically generated on a computer and used to determine the hardness, cohesiveness, and adhesiveness of the material. 

### 2.3. Rheological Properties

Rheological properties were assessed through swallow-related shear sweep, amplitude sweep, and frequency sweep tests at 25 °C using a rheometer (Physica MCR92, Anton Paar, Graz, Austria) with a plate-to-plate system (20 mm diameter, 1 mm gap) 24 h after processing.

The shear-dependent viscosity was measured over a shear rate range from 0.01 to 100 s^−1^ to determine the shear viscosity at 50 s^−1^ (η_50_).

The strain-dependent viscoelasticity was measured within the linear viscoelastic (LVE) region. An oscillation amplitude sweep test was conducted over a strain range from 0.01% to 1000% at a constant frequency of 1 Hz to assess the strain-related storage modulus (G′), loss modulus (G″), and loss tangent (tan δ).

The frequency-dependent viscoelasticity was measured within the linear viscoelastic (LVE) region. The storage (G′) and loss (G″) moduli were recorded across an angular frequency range from 0.1 to 100 rad s^−1^.

Shear recovery behavior was assessed using a three-step “low–high–low” flow sweep test. The test involved three flow peak holds: 0.1 s^−1^ for 3 min, 50 s^−1^ for 1 min, and 0.1 s^−1^ for 3 min.

The shear yield stress was determined by conducting a flow sweep over a shear rate from 0.001 to 0.1 s^−1^. The initial peak in shear stress was identified as the shear yield stress.

### 2.4. NDD and IDDSI Test

Testing of the National Dysphagia Diet (NDD) was conducted to classify the samples according to NDD levels. Shear-dependent viscosity was estimated across a shear rate from 0.01 to 100 s^−1^ to determine the shear viscosity at 50^−1^ (η_50_) [19].

International Dysphagia Diet Standardization Initiative (IDDSI) testing methods were used to categorize the samples according to the IDDSI levels. The fork pressure, fork separation, and finger test were employed [20].

### 2.5. Determination of Vitamins

Determination of water-soluble vitamins. Hydrogels were mixed with water (1:1 ratio), homogenized, and filtered through a 0.22 µm syringe filter. The filtrate was analyzed using a Nexera-i LC-2040C 3D Plus high-performance liquid chromatography system (Shimadzu corp., Kyoto, Japan) equipped with an Atlantis dC18 column (5 µm 4.6 × 150 mm; Waters, Ireland, Milford, MA, USA). Mobile phase A consisted of 0.1% TFA in H_2_O, and mobile phase B consisted of 0.1% TFA in acetonitrile. The time program for the gradient was as follows: B conc. 0% → 3% (0–5 min) → 15% (6 min) → 20% (10 min) → 100% (12 min) → 100% (25 min). Conditions: flow rate of 1.4 mL min^−1^, injection volume of 20 μL, column temperature at 30 °C. Detection was performed at 280 nm using a diode array detector.

Determination of vitamin D_3_. Samples were subjected to overnight saponification at 20 °C using KOH, ascorbic acid, and ethanol with continuous shaking. The mixture was then loaded onto a Chromabond XTR column (70 mL, 14.5 g; Macherey-Nagel GmbH & Co., Düren, Germany) and allowed to adsorb for 15 min. Vitamin D_3_ was eluted with 100 mL of n-hexane containing 5 mg of BHT (2,6-di-tert-butyl-p-cresol). The eluate was evaporated to dryness with a rotary evaporator (IKA RV 10, Staufen, Germany). The residue was reconstituted in 1 mL of mobile phase (acetonitrile: H_2_O, 100:5, *v/v*), filtered through a 0.22 µm filter, and analyzed by HPLC (Nexera-i LC-2040C 3D Plus; Shimadzu) using a Nucleodur C18 Isis column (5 µm, 2.0 × 125 mm; Macherey-Nagel). Conditions: flow rate of 0.2 mL min^−1^, column temperature at 25 °C, injection volume of 20 μL. Detection was carried out at 275 nm with a diode array detector.

Retention of vitamins during the processing was calculated using the following equation:

$$\mathrm{Retention}\;=\;\left(\left(C_{in}-C_{f}\right)/C_{in}\right)\times 100\%;$$

where $C_{in}$ is the amount of vitamin initially added to the hydrogel, and $C_{f}$ is the amount of vitamin measured after preparation.

Vitamin retention during storage was calculated using the same equation; however, in this case, $C_{in}$ represents the amount of vitamin measured immediately after hydrogel preparation, and *C_f_* represents the amount of vitamin measured after storage at 4 °C for 6, 16 and 24 weeks.

### 2.6. Vitamins Release During In Vitro Digestion

An aqueous micronutrient solution with the same vitamin content as the hydrogel was used as a control. The hydrogel was compressed to 50% of its initial height with a texture analyzer to simulate oral compression before digestion. After this, static in vitro digestion under conditions relevant to older individuals was performed according to the INFOGEST protocol [21,22]. Briefly, 5.0 g of the sample, mixed with 2.00 g of glass beads, was combined with 5 mL of simulated saliva (containing alpha amylase at 75 U/mL in the digesta) for 2 min. Next, it was combined with 10 mL of simulated gastric juice containing pepsin (1200 U/mL of digesta). The final digest volume was adjusted to 20 mL. After incubating for 3 h at 37 °C on an orbital shaker at 140 min^−1^ in a water bath (Thermolab, GFL 1092, Berlin, Germany), the intestinal phase was initiated by adding simulated intestinal fluids containing pancreatin (80 U/mL of digesta) and bile salts (6.7 mmol/L in the final digestion mixture) for 2 h. The total final volume of the sample was 40 mL. The target gastric pH was approximately 3.7, and the intestinal pH was maintained between 6.5 and 7. pH adjustments were analyzed at different digestion times: 0 min, 60 min, 120 min, and 180 min during the gastric stage (G0; G60; G120 and G180), as well as at 60 min and 120 min during the intestinal stage (D60, D120). Pepsin activity in the samples collected during the gastric phase was halted by adding NaOH until a pH of 7 was reached. All samples were cooled to 0–4 °C in ice water and centrifuged at 4000 rpm at +4 °C (MPW-260R, MPW Med. Instruments, Warsaw, Poland). After centrifugation, the samples were filtered, and the soluble fraction was collected, frozen, and stored at −18 °C until analysis. The digestion was performed twice. The vitamin content released into the gastrointestinal fluids was analyzed as described in the previous section. The vitamin release was calculated using the following equation:

$$\mathrm{Release}\;=\;\left(C_{f}/C_{in}\right)\times 100\%;$$

where $C_{in}$ is the amount of vitamin in the hydrogels, $C_{f}$ is the amount of vitamin measured in digesta.

### 2.7. Sensory Evaluation

Sensory evaluation was conducted at the Addere Care nursing and supportive care hospital in Lithuania (Ethical approval from the KTU Research Ethics Committee, 26 September 2024 No M6-2024-23). Informed consent was obtained from all patients involved in the study. The study included 62 participants (22 men and 40 women) with an average age of 78 years, ranging from 67 to 96 years old. Of these, 7 participants (11.3%) had dysphagia. Products were served at room temperature on a plate. Participants tasted each product and then completed a questionnaire with nine questions focusing on taste, comfort, and texture. They rated each question on a 7-point hedonic categorical scale with labels at each anchor, where 1 means “dislike extremely” and 7 “like extremely”. After evaluating each product, participants were asked to rinse their mouths with plain water.

### 2.8. Fiberoptic Endoscopic Evaluation of Swallowing (FEES)

Fiberoptic Endoscopic Evaluation of Swallowing was performed at the LSMU Kaunas Hospital Geriatrics Centre. The study was conducted in accordance with the Declaration of Helsinki, and approved by the Kaunas Regional Biomedical Research Ethics Committee (protocol code P1-BE-2-59/2018, date 11 October 2022). Informed consent was obtained from the patients involved in the study. A 3.7 mm HD Video Rhino-Laryngoscope (STORZ, Tuttlingen, Germany) was used. The FEES assessment was carried out according to the FEES Examination Protocol [23]. Aspiration risk was evaluated by using the Penetration–Aspiration Scale, PAS [24], pharyngeal residue was evaluated by using The Yale Pharyngeal Residue Severity Rating Scale, YPRSRS [25], and severity of oropharyngeal dysphagia by using Dysphagia Outcome and Severity Scale, DOSS [26]. The fluids were colored with food coloring. After the routine FEES procedure with water and thickened fluids, the patients were further examined using the Agar-CMC hydrogel.

### 2.9. Statistical Analysis

Unless otherwise stated, all of the experiments were conducted at least in duplicate and were statistically analyzed using one-way Analysis of Variance (ANOVA), and the average values were compared using Duncan’s multiple range test (*p <* 0.05). Statistical analyses were performed using SPSS 12.0 analysis software (StatSoft, Inc., Tulsa, OK, USA).

## 3. Results and Discussion

### 3.1. Appearance of Agar-CMC Hydrogels

**Figure A1:**
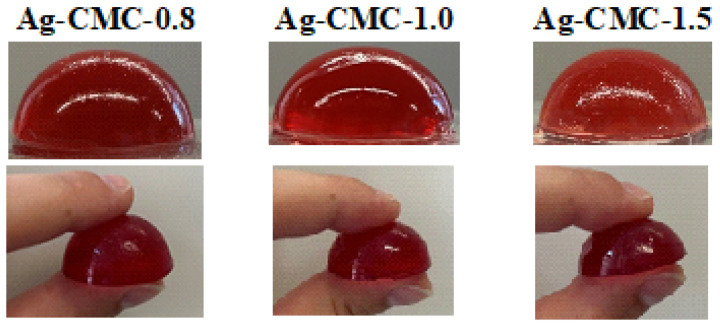
Photos of Agar-CMC hydrogels made with varying amounts of agar.

The applications and performance of Agar-CMC hydrogels as dysphagia-friendly, easy-to-handle, and transportable to the mouth depend on their shape and size. Figure A1 (Appendix A) presents photos of Agar-CMC hydrogels made with varying amounts of agar. All samples are self-supporting gels, hemispherical in shape, and one-bite, featuring a moderately thick interior and an elastic membrane covering the interior that resists breaking when lifted with fingers, yet melts in the mouth. They can be classified as “finger foods,” which refers to small, easily manageable food items that can be eaten without cutlery and are typically consumed by hand [7].

### 3.2. Rheological Characterization of Agar-CMC Hydrogels

The textural evaluation of Agar-CMC hydrogels showed that Ag concentration significantly affected hardness (*p* < 0.05), while it did not impact the adhesiveness or cohesiveness of the hydrogels (Table 1). Increasing agar concentration led to higher hardness in Agar-CMC hydrogels due to more hydrogen bonds and the aggregation of double helices into a three-dimensional network [27]. There is a direct relationship between gel hardness and the number of masticatory cycles, as well as the duration of oral processing needed for effective swallowing [28]. Low hardness values (144.3–368.15 g) suggest that Agar-CMC hydrogels can be easily bitten and squeezed between the tongue and palate. Low cohesiveness (0.12–0.13) helps prevent sudden disintegration during swallowing, ensuring safe swallowing. All hydrogels with low adhesiveness values (ranging from 10.61 ± 0.44 to 13.01 ± 0.54) require less time to form a suitable bolus, reducing the risk of choking during swallowing.

**Figure A2:**
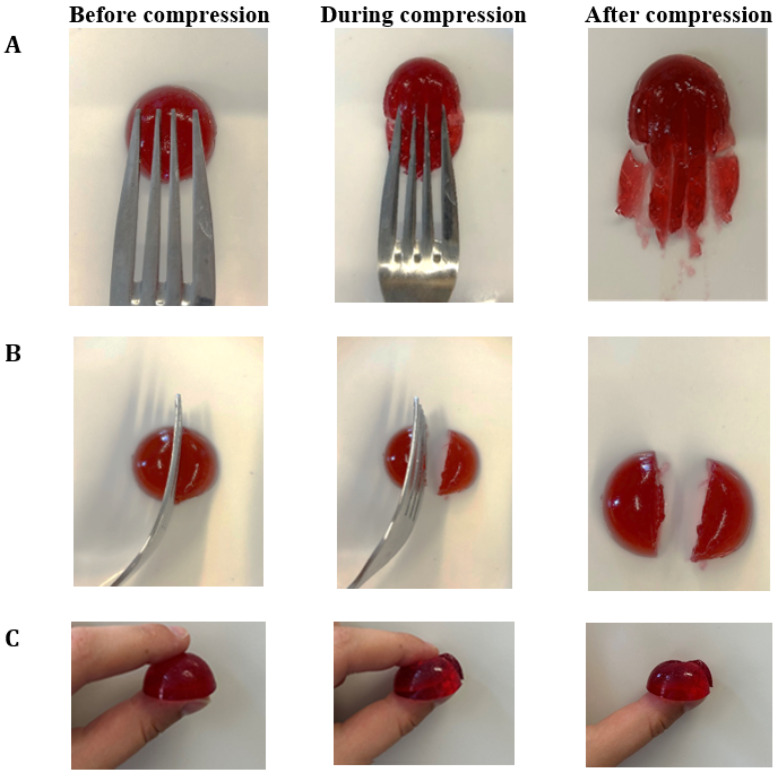
IDDSI Fork pressure test (**A**), Fork separation test (**B**) and Finger test (**C**) results for Agar-CMC-0.8.

**Figure A3:**
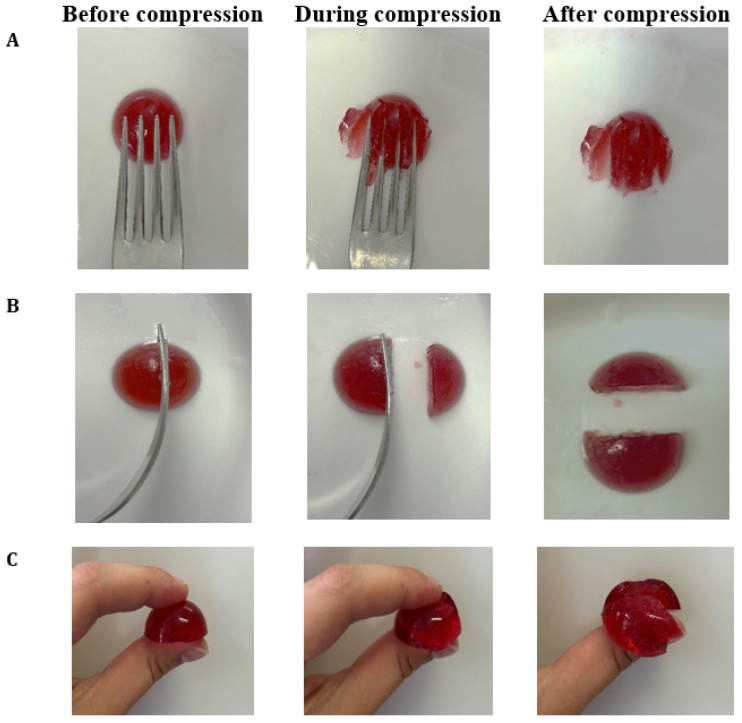
IDDSI Fork pressure test (**A**), Fork separation test (**B**) and Finger test (**C**) results for Agar-CMC-1.0.

**Figure A4:**
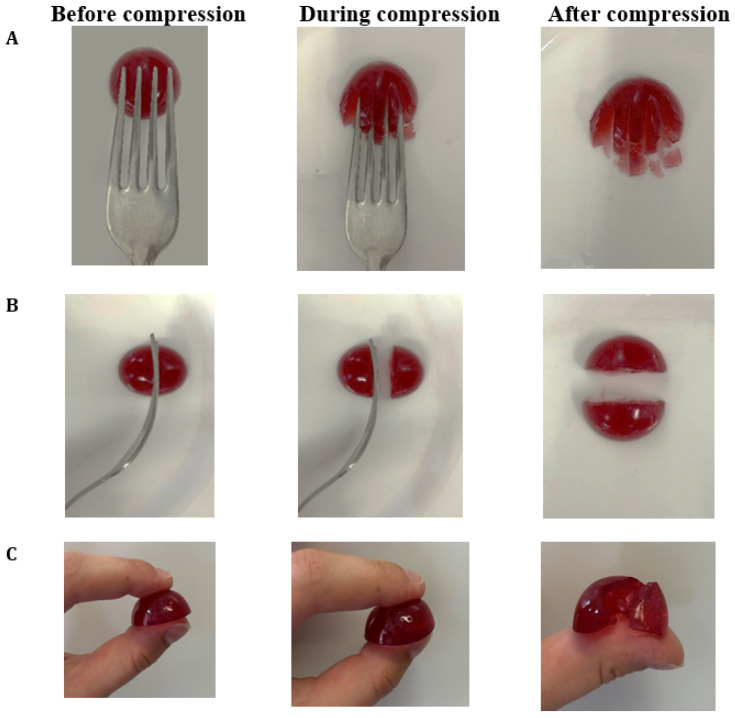
IDDSI Fork pressure test (**A**), Fork separation test (**B**) and Finger test (**C**) results for Agar-CMC-1.5.

The categorization of hydrogels within the NDD and IDDSI levels is shown in Table 1. As the agar concentration increased, the η_50_ values of hydrogels also increased. This classifies hydrogels with 0.8% agar as honey-like (350–1750 mPa·s), while those with 1.0% and 1.5% agar are categorized as spoon-thick or pudding-like (>1750 mPa·s) dysphagia-friendly products. The results of the fork pressure, fork separation, and finger tests were similar across all hydrogels (Figure A2, Figure A3 and Figure A4). When pressed with fork tines or a finger, they squashed, broke apart, and did not return to their original shape after the utensil was removed. Therefore, Agar-CMC hydrogels can be classified as level 6 soft and bite-sized foods according to IDDSI. This means they require chewing but not biting; they are tender and moist without visible liquid separation.

**Figure 1:**
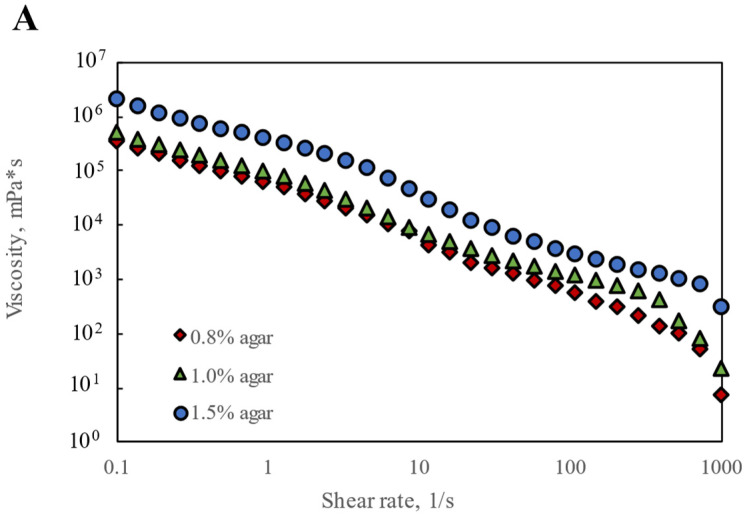

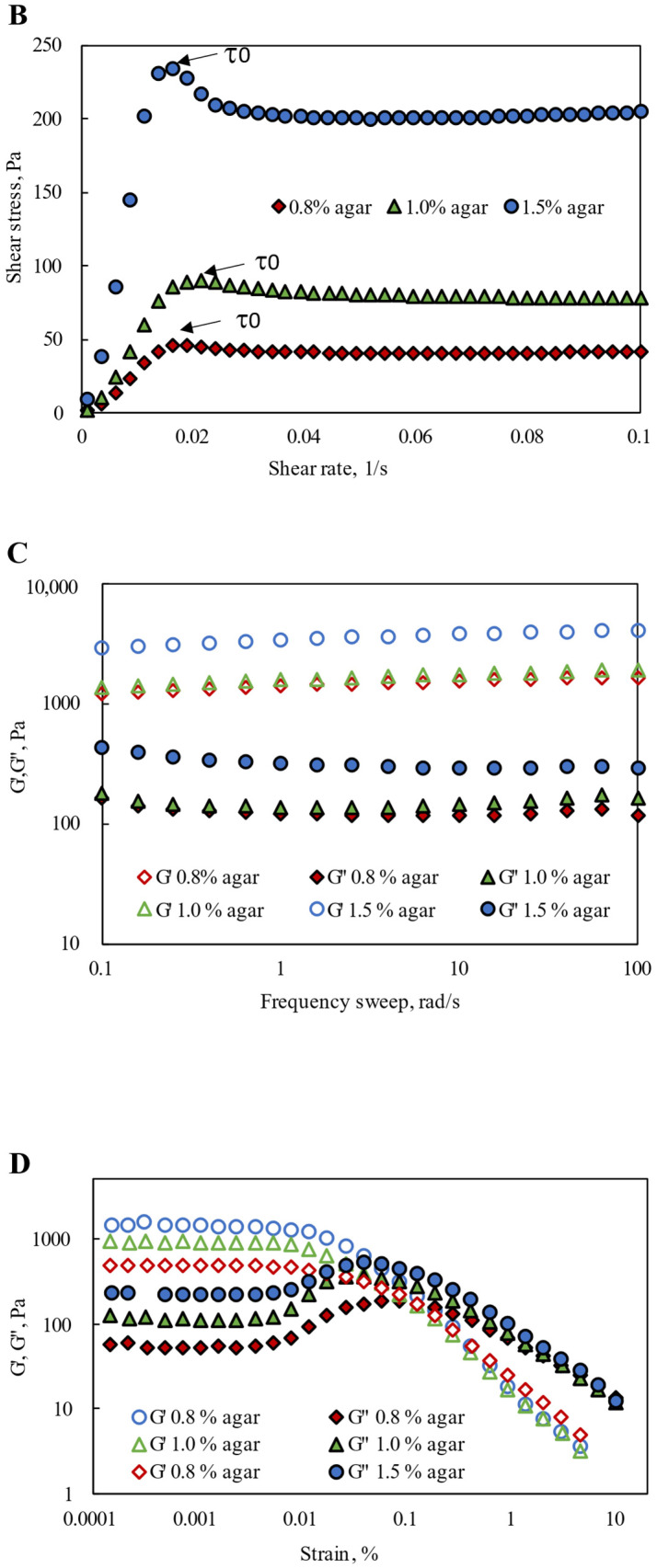

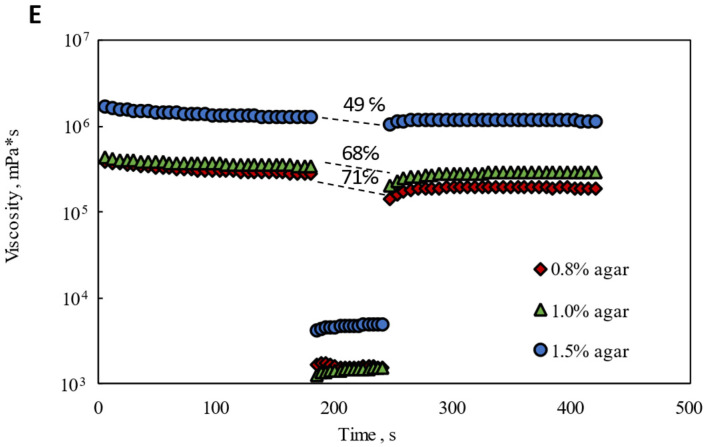
Rheological performance of Agar-CMC hydrogels: (**A**) Shear rate dependence of viscosity; (**B**) Shear yield stress; (**C**) Frequency dependence of G′ and G″; (**D**) Strain dependence of G′ and G″; (**E**) Thixotropic recovery.

Since Agar-CMC hydrogels have been found to exhibit textural properties that may facilitate easier and safer swallowing, their rheological performance during swallowing is extremely important. Shear sweep tests were conducted to gather evidence of the apparent shear flow behavior and shear yield stress of the hydrogels. All hydrogels displayed strong shear-thinning behavior, with viscosity decreasing as shear rate increased in the higher range of shear rates (0.01–100 s^−1^) (Figure 1A). This behavior is driven by the high molecular weight and polymeric structure of agar and carboxymethylcellulose—the polymer chains align in the flow direction, reducing internal resistance and viscosity [29]. An increase in apparent viscosity η_50_ was observed with rising agar concentration from 0.8% to 1.5%. Table 1 shows that Agar-CMC hydrogels with 1.0% and 1.5% agar exhibited a thickness level 4 according to the η_50_ range of NDD levels for thickening liquids (η_50_ > 1750 mPa s, pudding-like). This suggests they are suitable for people with severe dysphagia, considering that η_50_ corresponds to the shear rate in the oral cavity during swallowing.

The non-covalent interactions between agar and carboxymethylcellulose chains enable composite gels to act like elastic solids below a yield stress. When external forces on the gels exceed the internal forces that hold the gel structure together, the gels can flow freely. An increasing trend in shear yield stress was observed with higher agar concentrations in hydrogels, reaching up to 234.59 Pa at 1.5% agar at very low shear rates (0.001–0.1 s^−1^) (Figure 1B). Cuomo et al. [30] reported shear thinning behavior with a shear yield stress for chitosan and agar composite hydrogels, and this behavior was more noticeable at higher agar concentrations.

Agar-CMC hydrogels were further characterized through frequency- and strain-dependent dynamic viscoelastic tests. As the frequency increases and the motion speeds up, the hydrogel structure formed by the intertwined polymer molecules shows greater flexibility and stiffness. In this state, more deformation energy can be stored, although some is lost due to friction among molecules caused by reduced relative movement of their chains. Consequently, the elastic behavior (elastic modulus G′) becomes more dominant. At the same time, the proportion of lost deformation energy decreases, leading to a drop in the viscosity component (viscosity modulus G′) [31]. According to Figure 1C, all Agar-CMC hydrogels demonstrated significantly higher G‘ than G″, and both moduli remained relatively flat across a broad frequency range, indicating that, at rest, all samples are in a solid-state gel form. This finding aligns with IDDSI testing results, which classified all hydrogels as soft and one-bite size (Table 1). As the agar concentration increased, the elastic modulus G′ also increased, resulting in a stiffer structure in the hydrogels’ resting state. Hydrogels with 1.5% agar showed the stiffest and strongest gel structure. A higher agar concentration creates a denser three-dimensional network, further reinforced by the interconnected carboxymethyl cellulose network, which restricts the movement of polymer chains. These interactions reduce the number of flexible chains, which at lower agar concentrations tend to interact more frequently with each other and with water molecules. Additionally, increased network density reduces syneresis and improves water-binding efficiency within the hydrogel structure [32].

A deformation-viscoelasticity profile (amplitude sweep) was established, allowing for the identification of the LVR, within which G′ and G″ stay constant (Figure 1D). When the strain exceeded the LVR, the gel structure began to break down, indicating the gel’s transition from a solid to a liquid state. This feature is crucial for improving ease of swallowing. The length of the LVR slightly decreased as agar concentration in the hydrogel increased, suggesting that a slightly lower strain was needed for all hydrogels containing 1.5% agar to start a solid–fluid transition. A similar pattern is seen in the literature for agarose-based hydrogels, where higher polymer concentrations lead to higher elastic and viscous moduli, along with a notable reduction in both the LVR region and the LVE region threshold. These findings align with a higher network crosslinking density, resulting in a more organized polymer network with improved mechanical properties [33].

Finally, a three-step “low–high–low” test was performed to assess whether the Agar-CMC hydrogels tended to recover quickly after shearing during a simulated oral shearing process at a shear rate of 50 s^−1^ (Figure 1E). When the gels were exposed to a low, constant shear rate of 0.01 s^−1^ for 3 min, their viscosity slightly decreased. When subjected to a high, constant shear rate of 50 s^−1^, the viscosity suddenly dropped significantly and remained steady during this phase. Later, when the shear rate was reduced to 0.01 s^−1^ and maintained for 3 min, the viscosity of all hydrogels gradually recovered, though none returned to the initial viscosity. The shear recovery tests of Agar-CMC hydrogels showed a decreasing recovery factor in peak viscosity (from 75% to 49%) as agar concentration increased from 0.8% to 1.5%. It can be understood that higher agar concentration led to weaker interactions between gelling agent molecules. These weaker interactions contributed to solid-like behavior under lower yield stress and fluid behavior when these interactions were disrupted at higher yield stress. According to Zhang et al. [33], agarose hydrogels with higher concentration also had better recovery properties.

Texture profile analysis and rheology tests showed that increasing agar concentration in Agar-CMC hydrogels strengthens their internal structures, making them easy to pick up by hand, but the structure is easily broken during swallowing. The rheological properties of hydrogels depend on the competitive balance between gel formation kinetics and shear strength.

### 3.3. Suitability of Hydrogel for Vitamin Delivery and Controlled Release

**Figure 2:**
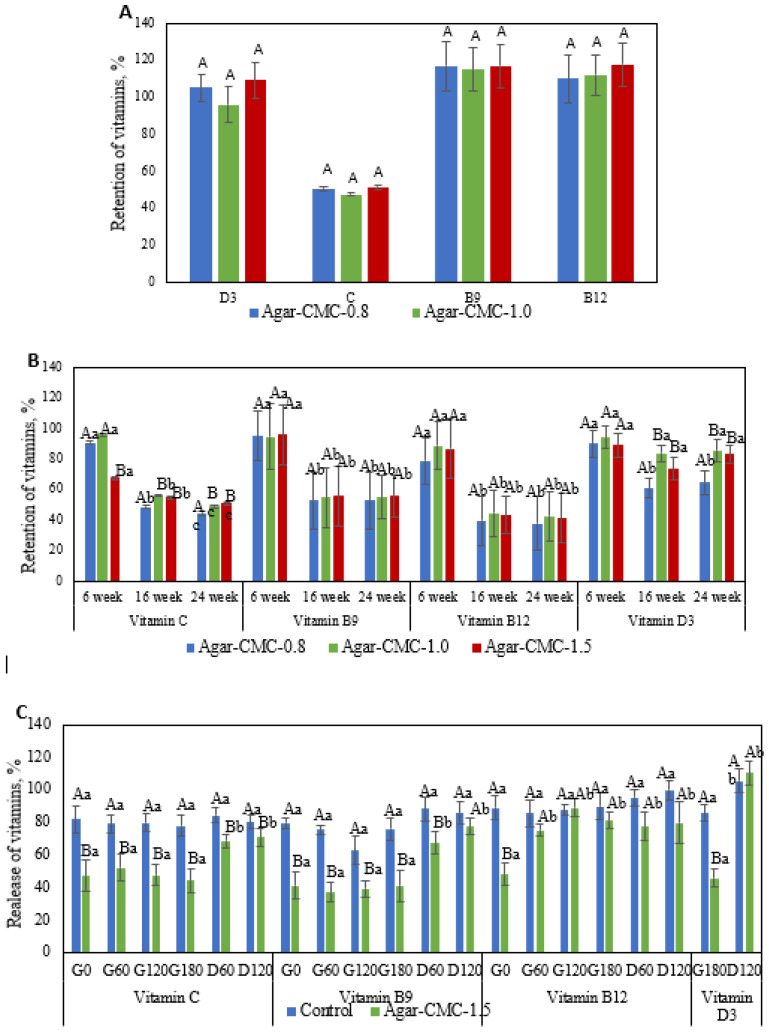
(**A**) Retention of added vitamins after processing. Upper case letters indicate significant (*p* < 0.05) differences in Agar-CMC hydrogels with different agar concentration; (**B**) Retention of added vitamins after six weeks of storage of Agar-CMC hydrogels at 4 °C. Lower case letters indicate significant (*p* < 0.05) differences in stability between different Agar-CMC hydrogels and upper case letters indicate significant (*p* < 0.05) differences in Agar-CMC hydrogels stability during storage; (**C**) Release kinetics of vitamins during in vitro digestion of Agar-CMC hydrogels. Lower case letters indicate significant (*p* < 0.05) differences in vitamin release at different stages of in vitro digestion and upper case letters indicate significant (*p* < 0.05) differences in vitamin release between control and Agar-CMC hydrogel.

The three-dimensional network structure of hydrogels makes them popular systems for delivering bioactive compounds [34]. To benefit from Agar-CMC hydrogels loaded with vitamins, these compounds must remain stable during manufacturing and have a suitable shelf life. This study evaluated the retention of added vitamins after processing and six weeks of storage at 4 °C to assess their stability (Figure 2A,B). The lowest retention after processing was observed for vitamin C, which is highly sensitive to degradation caused by heat, light, oxygen, and extreme pH levels, leading to rapid loss of activity during both processing and storage [35]. Agar-CMC hydrogels effectively retained vitamins B_9_, B_12_, and D_3_. A similar finding was previously reported, indicating that adding CMC to the gelled water phase of a double emulsion results in higher retention rates for both vitamin B_12_ and vitamin D_3_ [36]. Incorporating vitamins C, B_9_, B_12_, and D_3_ into the hydrogel’s structure protected them during six weeks of storage (Figure 2B). Over extended storage, vitamins C, B_9_, and B_12_ experienced more degradation than vitamin D_3_. Our results did not show a statistically significant (*p* < 0.05) effect of CMC content in the hydrogel on vitamin stability during storage.

When exposed to specific environmental conditions (e.g., pH, ionic strength, temperature, enzymes, etc.), hydrogels can disintegrate, which benefits targeted delivery of bioactives [17]. The release kinetics of vitamins added to Agar-CMC-1.5 during in vitro digestion were measured. These data are shown in Figure 2C as the ratio of the amount of individual vitamins found in the liquid digestive phase to the amount of vitamin in the hydrogel. To assess how gel structure affects the release kinetics of vitamins, a control non-gelled formulation was used. During the digestion period of the hydrogel and the non-gelled control, significant differences were observed in the release of all added vitamins. Vitamin release started immediately after gastric fluids (G0) were introduced to the samples and remained steady throughout the gastric phase. At the beginning of the intestinal phase (D60), there was a noticeable increase in vitamin release, but additional incubation with intestinal fluids did not impact vitamin release. By the end of the duodenal phase, 70–100% of the vitamins had been released.

However, the release kinetics of vitamins during in vitro digestion differ between gelled and non-gelled samples. Under gastric conditions, non-gelled samples showed a significantly higher rate of vitamin release compared to gelled samples. At G180, 77.9 ± 6.4% of the added vitamin C was released from the control sample, while 44.1 ± 7.3% was released from the gelled sample. By the end of the intestinal phase (D120), the amount of vitamin C released from the non-gelled samples was 80.2 ± 4.4%, whereas the gelled sample released 70.7 ± 5.2%. The release patterns of vitamins B_9_ and B_12_ followed these same trends. These findings are consistent with previous studies [17] on the release of vitamin C from a novel polyelectrolyte complex hydrogel formed through the self-assembly of salecan and chitosan; the release amount in simulated intestinal fluid was significantly higher than in simulated gastric fluid. During the gastric phase, 85.6 ± 4.9% and 45.7 ± 5.5% of vitamin D_3_ were released from the non-gelled and gelled samples, respectively. The release of this vitamin continued to increase during the intestinal phase, reaching nearly 100% at the end of digestion for both samples. Similarly, the formulation containing sodium alginate exhibited controlled release of vitamin D_3_ under simulated gastric conditions, with the highest overall release observed under simulated intestinal conditions [36]. These results suggest that the gel structure may restrict the excretion of vitamins C, B_9_, B_12_, and D_3_ during the gastric stage, while allowing for 70–100% release of these vitamins during intestinal digestion.

### 3.4. Sensory Acceptability and Swallowing Safety

Eating with hands is essential for older people who struggle with cutlery (apraxia) or find it hard to stay at the table during a meal. People with apraxia often use their fingers to eat, even when the food’s shape is not suitable [37]. The sensory analysis results for Agar-CMC-1.5 are shown in Table 2. The findings suggest that the hydrogel was easily picked up by hand, with 51.6% of respondents giving it the highest score (7/7). Participants rated the mouthfeel with scores of 5 points or higher. In evaluating the softness of the hydrogel, results indicated that 40% of respondents described it as very juicy. Survey results also showed that the hydrogels are easy to swallow, with an average score of 6.23 out of 7. Participants with dysphagia rated the ease of swallowing the hydrogel at an average of 6.5. The aroma intensity and aftertaste were considered acceptable, with evaluators giving an average score of 3–4 points. The property related to the instrumental method—adhesiveness—refers to the mouthfeel, which was also rated as uncharacteristic for this hydrogel, receiving about 6 points (with 7 meaning no mouthfeel at all).

During the initial phase of this study, we examined changes in the rheological properties of Agar-CMC hydrogels during swallowing and evaluated their suitability for people with dysphagia. In the final phase, we conducted a fiberoptic endoscopic swallowing case study of the hydrogels.

Case study. A 78-year-old patient with unknown cause of dysphagia was examined by FEES. It was determined that the mucous membrane of this patient was smooth, pink in color, and slight accumulations of saliva were noted on the arytenoids and in the pyriform sinuses. The posterior pharyngeal wall was symmetrical, but the vocal folds were slightly asymmetrical—the right side was thinner and did not close completely. Sensitivity (evaluated by touch test) was slightly impaired. Swallowing movements were slightly weakened, but the strength of the tongue remained adequate. At the beginning of the study, the patient was given 5 mL of water to drink. During swallowing, there was no “white out”; the liquid flowed onto the epiglottis, arytenoids, and into the pyriform sinuses; pharyngeal residue was evaluated 2 points on YPRSRS. The voice changed, and the patient coughed—signs of aspiration were observed.

Using the thickener “Nutilis Clear” (Nutricia Ltd., Trowbridge, UK), a liquid was prepared to achieve level 2 according to the IDDSI classification. The patient was given 10 mL of the thickened liquid. During swallowing, the “white out” was completely absent, and the content entered the epiglottis, epiglottic valleculae, and pyriform sinuses, but no signs of aspiration were observed, nor was there any cough. After the third swallow, the content was gone, and the sinuses and valleculae cleared. Then 10 mL of a liquid with the consistency level 3 according to IDDSI was used, during which penetration occurred; the content spilled onto the epiglottis, and about half remained in the valleculae and sinuses after swallowing. Swallowing was needed 2–3 times. When drinking a liquid with viscosity level 4 according to IDDSI (10 mL) during initiation of swallowing, the content spilled onto the epiglottis, valleculae, pyriform sinuses, and roughly half remained in the valleculae and sinuses afterward the first swallowing. Swallowing was required 2 times. The patient was diagnosed with mild to moderate oropharyngeal dysphagia. A high aspiration risk was identified (PAS 6 points) when using unmodified liquids, and a low risk (PAS 3 points) when using modified liquids [24].

**Figure 3:**
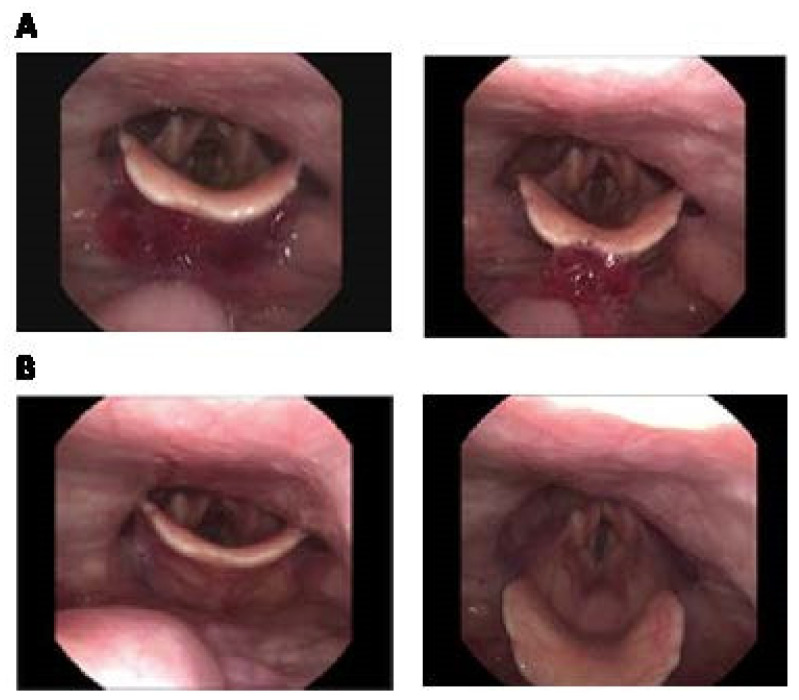
Fiberoptic Endoscopic Evaluation of Swallowing photos of Agar-CMC-1.5 hydrogel. (**A**) during the initiation of swallowing: there are no “white out” and the content is visible in the valleculae and on the epiglottis; (**B**) after swallowing: no residue in the valleculae and in the pyriform sinus (1 point in the Yale Pharyngeal Residue Severity Rating Scale, YPRSRS).

The study involved administering the patient with Agar-CMC-1.5 hydrogel. During swallowing, the “white out” was partially absent; delayed initiation of swallowing was observed, and the chewed bolus fell in the valleculae and onto the epiglottis (Figure 3A). The patient swallowed the content and there were no signs of aspiration with no residues remaining in the valleculae or pyriform sinuses (Figure 3B). The patient found the hydrogel easy to chew and comfortable to swallow. According to the Penetration-Aspiration scale, the aspiration risk when consuming Agar-CMC hydrogels was rated at 1 PAS point.

## 4. Conclusions

Hydrogels were created from a mixture of agar and carboxymethylcellulose (Agar-CMC), with the carboxymethylcellulose content kept constant at 0.5%, while the agar content was varied at 0.8%, 1.0%, and 1.5%. The texture and rheological properties of these hydrogels confirmed their suitability for forming water-rich, shape-retaining structures that provide nutritional support to elderly individuals with dysphagia. According to the IDDSI classification, all Agar-CMC hydrogels were rated as level 6 soft and bite-sized foods, indicating they require chewing but not biting; they are tender and moist, with no visible liquid separation. During NDD tests, when viscosity was measured at a shear rate of 50 s^−1^, hydrogels with 0.8% agar were classified as honey-thick, while those with 1.0% and 1.5% agar were classified as spoon-thick. Shear-thinning behavior and a shear yield stress were observed in the Agar-CMC hydrogels, with this behavior becoming more pronounced at higher agar concentrations. Dynamic viscoelastic tests revealed a higher network crosslinking density in hydrogels with increased agar concentration, resulting in a more organized polymer network with enhanced mechanical and recovery properties. Hydrogels with 1.5% agar showed the stiffest and strongest gel structure, the highest yield stress and the lowest recovery factor in peak viscosity in comparison with hydrogels containing 0.8 and 1.0% of agar. Incorporating vitamins C, B_9_, B_12_, and D_3_ into the hydrogel structure protected them during processing and six weeks of storage. Vitamin C showed the lowest retention rate—47.5% to 51.4%—while the decline in vitamins B_9_, B_12_, and D_3_ was not statistically significant (*p* > 0.05). Over prolonged storage, vitamins C, B_12_, and B_9_ experienced more degradation than vitamin D_3_; however, the hydrogel’s CMC content did not have a statistically significant (*p* < 0.05) effect on vitamin stability during storage. In vitro digestion results suggest that the gel structure limited the excretion of vitamins C, B_9_, B_12_, and D_3_ during the gastric phase, while allowing 70–100% release of these vitamins during intestinal digestion. Finally, sensory evaluation (*n* = 62) and endoscopic swallowing evaluation of Agar-CMC hydrogels demonstrated that they are suitable and safe for individuals with mild to moderate oropharyngeal dysphagia. They were easy to handle, soft, and juicy, and did not cause unpleasant sensations in the mouth or during swallowing. Future research is needed for the optimization of polysaccharides concentrations in the composite hydrogels and their more extensive testing with dysphagia patients.

## Figures and Tables

**Table 1:** Textural properties of Agar-CMC hydrogels and their categorization within the NDD and IDDSI levels.

Agar Content in Agar-CMC Hydrogels, %	Textural Properties *			Classification as Products Indicated for People with Dysphagia	
	Hardness, g	Adhesion, g.s	Cohesiveness	NDD ** Testing Results	IDDSI *** Testing Results
0.8	144.3 ± 10.13A	−13.01 ± 0.54A	0.12 ± 0.01A	Level III (η_50_ = 1096.21 mPa∙s), honey-like	Level 6, soft, one bite size, views in Figure A2 (Appendix A)
1.0.	187.47 ± 31.48B	−11.79 ± 0.92A	0.13 ± 0.01A	Level IV (η_50_ = 1949.85 mPa∙s), pudding-like	Level 6, soft, one bite size, views in Figure A3 (Appendix A)
1.5	368.15 ± 59.05C	−10.61 ± 0.44B	0.12 ± 0.01A	Level IV (η_50_ = 5538.25 mPa∙s), pudding-like	Level 6, soft, one bite size, views in Figure A4 (Appendix A)

* Values are presented as mean ± standard deviation. Uppercase letters indicate significant (*p* < 0.05) differences in the characteristics of Agar-CMC hydrogels with different agar content. ** The National Dysphagia Diet. *** The International Dysphagia Diet Standardisation Initiative.

**Table 2:** Results of sensory evaluation of Agar-CMC-1.5.

Question	Evaluation
Difficult to take by hand	6.00 ± 1.34
Liking of the color	6.03 ± 1.38
Liking of the mouth feeling	5.50 ± 1.83
The intensity of the taste and aroma	2.67 ± 1.83
Firmness	5.08 ± 1.79
Juiciness	4.28 ± 1.81
Swallowing difficulty	6.23 ± 1.42
Afterfeel intensity	3.93 ± 1.89
Mouth counting after-feel	5.82 ± 1.60

## Data Availability

Data is contained within the article.

## References

[1] Thiyagalingam S., Kulinski A.E., Thorsteindottir B., Schindelar K.L., Takahashi P.Y. (2021). Dysphagia in older adults. Mayo Clin. Proc..

[2] Viñas P., Bolívar-Prados M., Tomsen N., Costa A., Marin S., Barcons N., Clavé P. (2023). Prevalence of dehydration among adult patients with Oropharyngeal Dysphagia: A systematic and scoping review. Clin. Nutr. ESPEN.

[3] O’Keeffe S.T. (2018). Use of modified diets to prevent aspiration in oropharyngeal dysphagia: Is current practice justified?. BMC Geriatr..

[4] Newman R., Vilardell N., Clavé P., Speyer R. (2016). Effect of Bolus Viscosity on the Safety and Efficacy of Swallowing and the Kinematics of the Swallow Response in Patients with Oropharyngeal Dysphagia: White Paper by the European Society for Swallowing Disorders (ESSD). Dysphagia.

[5] Vivanti A.P., Campbell K.L., Suter M.S., Hannan-Jones M.T., Hulcombe J.A. (2009). Contribution of thickened drinks, food and enteral and parenteral fluids to fluid intake in hospitalised patients with dysphagia. J. Hum. Nutr. Diet..

[6] Pouyet V., Giboreau A., Benattar L., Cuvelier G. (2014). Attractiveness and consumption of finger foods in elderly Alzheimer’s disease patients. Food Qual. Prefer..

[7] Forsberg S., Nyberg M., Olsson V., Rothenberg E., Bredie W.L.P., Wendin K., Westergren A. (2024). Finger Food Meals as a Means of Improving Mealtimes for People with Motoric Eating Difficulties: A Pilot Study. J. Nutr. Gerontol. Geriatr..

[8] Murphy J.L., Holmes J., Brooks C. (2017). Nutrition and dementia care: Developing an evidence-based model for nutritional care in nursing homes. BMC Geriatr..

[9] Romito L.M. (2003). Introduction to nutrition and oral health. Dent. Clin. N. Am..

[10] DiMaria-Ghalili R.A. (2014). Integrating Nutrition in the Comprehensive Geriatric Assessment. Nutr. Clin. Pract..

[11] ter Borg S., Verlaan S., Hemsworth J., Mijnarends D.M., Schols J.M., Luiking Y.C., de Groot L.C. (2015). Micronutrient intakes and potential inadequacies of community-dwelling older adults: A systematic review. Br. J. Nutr..

[12] Gomollón F., Dignass A., Annese V., Tilg H., Van Assche G., Lindsay J.O., Peyrin-Biroulet L., Cullen G.J., Daperno M., Kucharzik T. (2017). 3rd European Evidence-based Consensus on the Diagnosis and Management of Crohn’s Disease 2016: Part 1: Diagnosis and Medical Management. J. Crohn’s Colitis.

[13] Sharma S., Bhende M., Goel A. (2024). A review: Polysaccharide-based hydrogels and their biomedical applications. Polym. Bull..

[14] Siddiqui S.A., Alvi T., Biswas A., Shityakov S., Gusinskaia T., Lavrentev F., Dutta K., Khan M.K.I., Stephen J., Radhakrishna M. (2022). Food gels: Principles, interaction mechanisms and its microstructure. Crit. Rev. Food Sci. Nutr..

[15] Mikula K., Izydorczyk G., Mironiuk M., Szopa D., Chojnacka K., Witek-Krowiak A. (2024). Selection of matrix material for hydrogel carriers of plant micronutrients. J. Mater. Sci..

[16] Boughriba S., Souissi N., Nasri R., Nasri M., Li S. (2021). pH sensitive composite hydrogels based on gelatin and reinforced with cellulose microcrystals: In depth physicochemical and microstructural analyses for controlled release of vitamin B_2_. Mater. Today Commun..

[17] Hu X., Wang Y., Zhang L., Xu M. (2020). Formation of self-assembled polyelectrolyte complex hydrogel derived from salecan and chitosan for sustained release of Vitamin C. Carbohydr. Polym..

[18] Yang Z., McClements D.J., Li C., Sang S., Chen L., Long J., Qiu C., Jin Z. (2023). Targeted delivery of hydrogels in human gastrointestinal tract: A review. Food Hydrocoll..

[19] American Dietetic Association (2002). National Dysphagia Diet: Standardization for Optimal Care.

[20] IDDSI, Framework Testing Methods 2.0. https://www.iddsi.org/images/Publications-Resources/DetailedDefnTestMethods/English/V2TestingMethodsEnglish31july2019.pdf.

[21] Brodkorb A., Egger L., Alminger M., Alvito P., Assunção R., Ballance S., Bohn T., Bourlieu-Lacanal C., Boutrou R., Carrière F. (2019). INFOGEST static in vitro simulation of gastrointestinal food digestion. Nat. Protoc..

[22] Menard O., Lesmes U., Shani-Levi C.S., Araiza Calahorra A., Lavoisier A., Morzel M., Rieder A., Feron G., Nebbia S., Mashiah L. (2023). Static in vitro digestion model adapted to the general older adult population: An INFOGEST international consensus. Food Funct..

[23] Langmore S. (2000). Endoscopic Evaluation and Treatment of Swallowing Disorders.

[24] Rosenbek J.C., Robbins J.A., Roecker E.B., Coyle J.L., Wood J.L. (1996). A Penetration-Aspiration Scale. Dysphagia.

[25] Neubauer P.D., Rademaker A.W., Leder S.B. (2015). The Yale pharyngeal residue severity rating scale: An anatomically defined and imagebased tool. Dysphagia.

[26] O’Neil K.H., Purdy M., Falk J., Gallo L. (1999). The dysphagia outcome and severity scale. Dysphagia.

[27] Smirnov V., Khramova D., Chistiakova E., Zueva N., Vityazev F., Velskaya I., Popov S. (2024). Texture Perception and Chewing of Agar Gel by People with Different Sensitivity to Hardness. Gels.

[28] Park Y.S., Hong H.P., Ryu S.R., Lee S., Shin W.S. (2022). Effects of textured food masticatory performance in older people with different dental conditions. BMC Geriatr..

[29] El-hefian E.A., Yahaya A.H. (2010). Effects of temperature, shearing time and rate of shear on the viscosity of chitosan/agar-blend solutions. Maejo Int. J. Sci. Technol..

[30] Cuomo F., Cofelice M., Lopez F. (2019). Rheological Characterization of Hydrogels from Alginate-Based Nanodispersion. Polymers.

[31] Stojkov G., Niyazov Z., Picchioni F., Bose R.K. (2021). Relationship between Structure and Rheology of Hydrogels for Various Applications. Gels.

[32] Cofelice M., Messia M.C., Marconi E., Cuomo F., Lopez F. (2023). Effect of the xanthan gum on the rheological properties of alginate hydrogels. Food Hydrocoll..

[33] Zhang H., Guo H., Liu Y., Shi C., Pan L., Zhang X., Zou J. (2023). Thixotropic composite hydrogels based on agarose and inorganic hybrid gellants. Chin. J. Chem. Eng..

[34] Li J., Jia X., Yin L. (2021). Hydrogel: Diversity of Structures and Applications in Food Science. Food Rev. Int..

[35] Giannakourou M.C., Taoukis P.S. (2021). Effect of Alternative Preservation Steps and Storage on Vitamin C Stability in Fruit and Vegetable Products: Critical Review and Kinetic Modelling Approaches. Foods.

[36] He L., Hu S., Zhang G., Wang X., Zhao Y., Wang Q., Liu M., Wang Z., Sangeeta P., Ding Z. (2024). Influence of polysaccharide-based co-encapsulants on efficiency, stability, and release of vitamins B12 and D3 in multilayered microcapsules. J. Food Eng..

[37] Forsberg S., Olsson V., Bredie W.L.P., Wendin K. (2022). Vegetable finger foods—Preferences among older adults with motoric eating difficulties. Int. J. Gastron. Food Sci..

